# Warm Autoimmune Hemolytic Anemia as a Rare Presentation of Pre-fibrotic Primary Myelofibrosis

**DOI:** 10.7759/cureus.75155

**Published:** 2024-12-05

**Authors:** Steven Y Lai, Amanda E Lo, Phillis Wu

**Affiliations:** 1 Hematology and Oncology, Olive View University of California Los Angeles (UCLA) Medical Center, Sylmar, USA; 2 Pathology, Olive View University of California Los Angeles (UCLA) Medical Center, Sylmar, USA

**Keywords:** calreticulin (calr), essential thrombocythemia, pre-fibrotic myelofibrosis, primary myelofibrosis, warm autoimmune hemolytic anemia

## Abstract

Primary myelofibrosis (PMF) is an uncommon chronic myeloproliferative disorder that is commonly associated with Janus kinase 2 (JAK-2), calreticulin (CALR), or thrombopoietin receptor (MPL) mutations. Pre-fibrotic PMF (also known as pre-PMF or early PMF) is a subtype of PMF that is defined by a lower grade of fibrosis. In this report, we present a rare case of warm autoimmune hemolytic anemia (wAIHA) associated with pre-PMF.

The patient is a 64-year-old female with chronic thrombocytosis who presented with wAIHA. She underwent treatment with high-dose steroids and intravenous immunoglobulin which was unsuccessful. Subsequent treatment with rituximab successfully caused her wAIHA to go into complete remission, without the need for maintenance therapy.

A bone marrow biopsy showed pre-PMF. Her warm autoimmune hemolytic anemia was thought to be related to cytokine dysregulation and autoantibody formation, which is commonly seen in pre-PMF.

Underlying myelofibrosis should be considered in a patient who presents with wAIHA and other hematologic abnormalities. In such cases, a bone marrow biopsy should be performed as part of the workup. Further investigation is required to determine the mechanism behind this association.

## Introduction

Primary myelofibrosis (PMF) is a rare chronic myeloproliferative disorder that is commonly associated with Janus kinase 2 (JAK-2), calreticulin (CALR), or thrombopoietin receptor (MPL) mutations [1]. In this report, we discuss a patient who presented with warm autoimmune hemolytic anemia (wAIHA) and was subsequently found to have pre-fibrotic primary myelofibrosis (also known as pre-PMF or early PMF). Her warm autoimmune hemolytic anemia was thought to be related to cytokine dysregulation and autoantibody formation in pre-PMF.

## Case presentation

A 64-year-old Ukrainian female was initially referred to a hematology clinic for evaluation of thrombocytosis. Her past medical history included hypertension, hyperlipidemia, hypothyroidism, and solitary kidney due to trauma. Complete blood count (CBC) was significant for mild thrombocytosis and anemia (See Table 1 for corresponding lab values). White blood cells were within normal limits with unremarkable differential. A thrombocytosis gene mutation panel was positive for CALR mutation but was negative for both JAK-2 and MPL mutations. Iron panel, folate, and vitamin B12 levels were all within normal limits. Unfortunately, the patient was lost to follow-up before further workup could be completed.

**Table 1:** Corresponding lab values of hemoglobin, platelets, and hemolysis chronologically IVIG: intravenous immunoglobulin

Date	Hemoglobin (g/dL, reference range 12.0-14.6)	Platelets (thousand/mm3, reference range 160-360)	Haptoglobin (mg/dL, reference range 36-195)	Retic Index (>2 indicates adequate bone marrow response to degree of anemia)	Bilirubin (mg/mL, reference range 0.1-1.2)	LDH (units/L, reference range 98-192)
Initial presentation to hematology clinic	11.4	646	212	2.2	0.8	N/A
Four years after initial presentation	6.3	568	N/A	N/A	1.7	N/A
In clinic (Hospital Day 0)	6.5	673	<15	4.8	2.7	561
Hospital day 1 (started steroids)	5.5	561	<15	3.3	2.9	484
Hospital day 2 (started IVIG)	6.3	586	<15	3.5	2.1	N/A
Hospital day 7 (started rituximab)	6.2	619	<15	3.7	1.3	512
Hospital day 8	7.9	637	<15	5.3	1.4	491
Hospital day 9 (discharged)	8.1	637	<15	7.2	1.4	420
One week after discharge	10.6	694	76	N/A	0.7	344
Three weeks after discharge (last dose of rituximab)	11.4	1061	111	3.2	0.7	370
Two months after discharge (last dose of steroids)	11.8	649	143	2.1	0.8	463
Three months after discharge	12.3	540	123	1.5	0.6	404
One year after discharge	12.4	260	107	2.8	1.1	473

Four years later, she was referred again to a hematology clinic due to worsening anemia. At her hematology clinic appointment, she complained of fatigue and muscle pain for the past two months. Vitals were within normal limits. Physical examination revealed pallor and palpable splenomegaly. CBC showed critically low hemoglobin with undetectable haptoglobin. Thrombocytosis was stable. Reticulocyte index, bilirubin, and lactate dehydrogenase (LDH) were all elevated. The patient was instructed to present to the emergency department due to concern for hemolytic anemia.

Direct antibody test (DAT) was consistent with wAIHA based on the presence of polyspecific, anti-immunoglobulin G (IgG), and anti-complement 3 (C3) antibodies. Repeat CBC showed worsening hemoglobin. The patient was subsequently admitted to the hospital and started on prednisone 1mg/kg daily, as well as intravenous immunoglobulin (IVIG) 0.4 g/kg followed by 1 g/kg for the following two days.

Despite these interventions, the patient continued to have hemolysis, with unchanged hemoglobin levels and undetectable haptoglobin. As a result, she was given rituximab 375 mg/m^2^ on hospital day 7. The patient showed improvement in her hemoglobin and was discharged on hospital day 9. She subsequently received three additional treatments of weekly rituximab and a two-month prednisone taper as an outpatient. One week after discharge, the patient’s haptoglobin returned to normal. Three months later, her hemoglobin reached normal levels.

**Figure 1:**
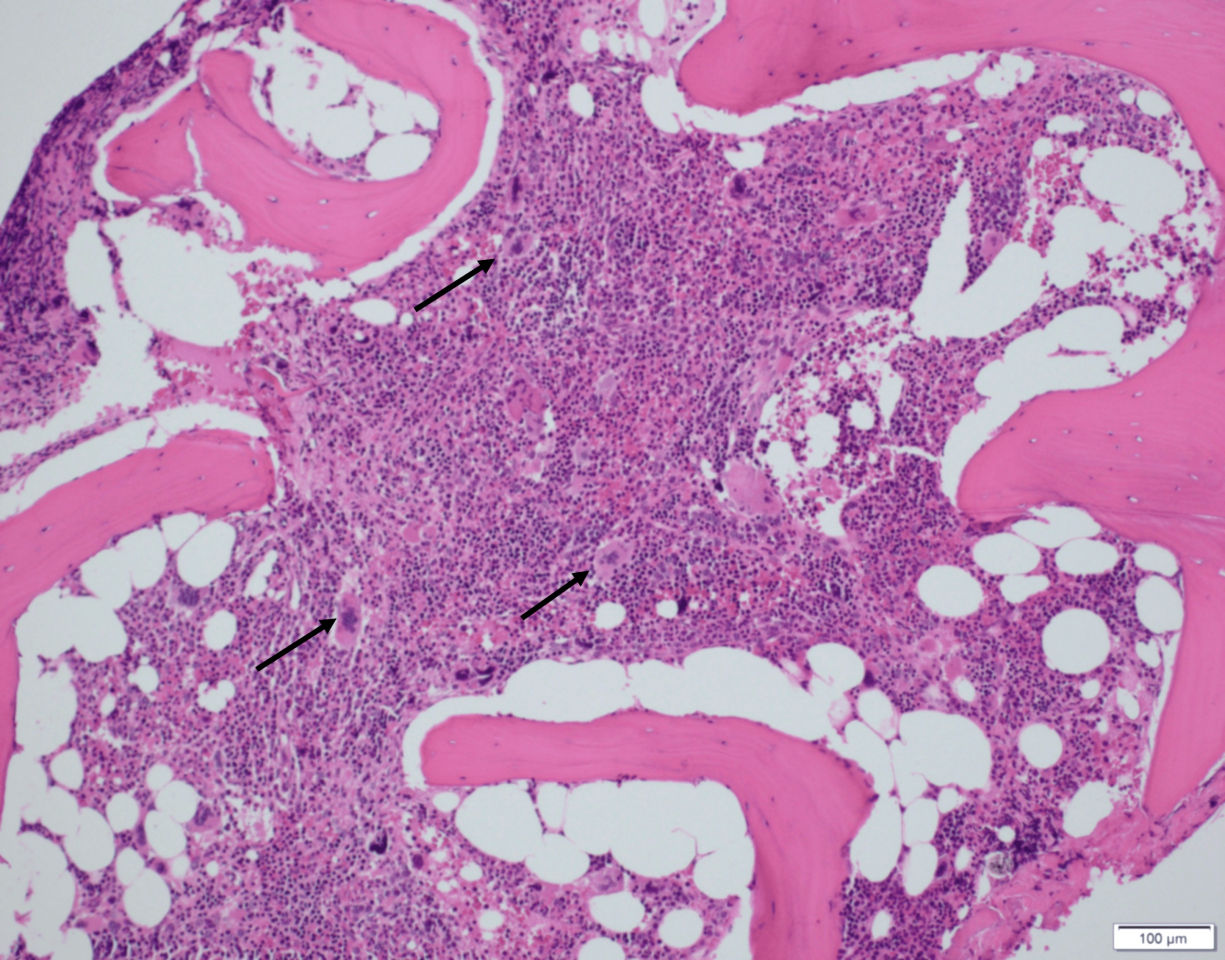
Bone core biopsy with hypercellular marrow, with atypical megakaryocytic proliferation shown by arrows. Hematoxylin and eosin staining; magnification ×100

**Figure 2:**
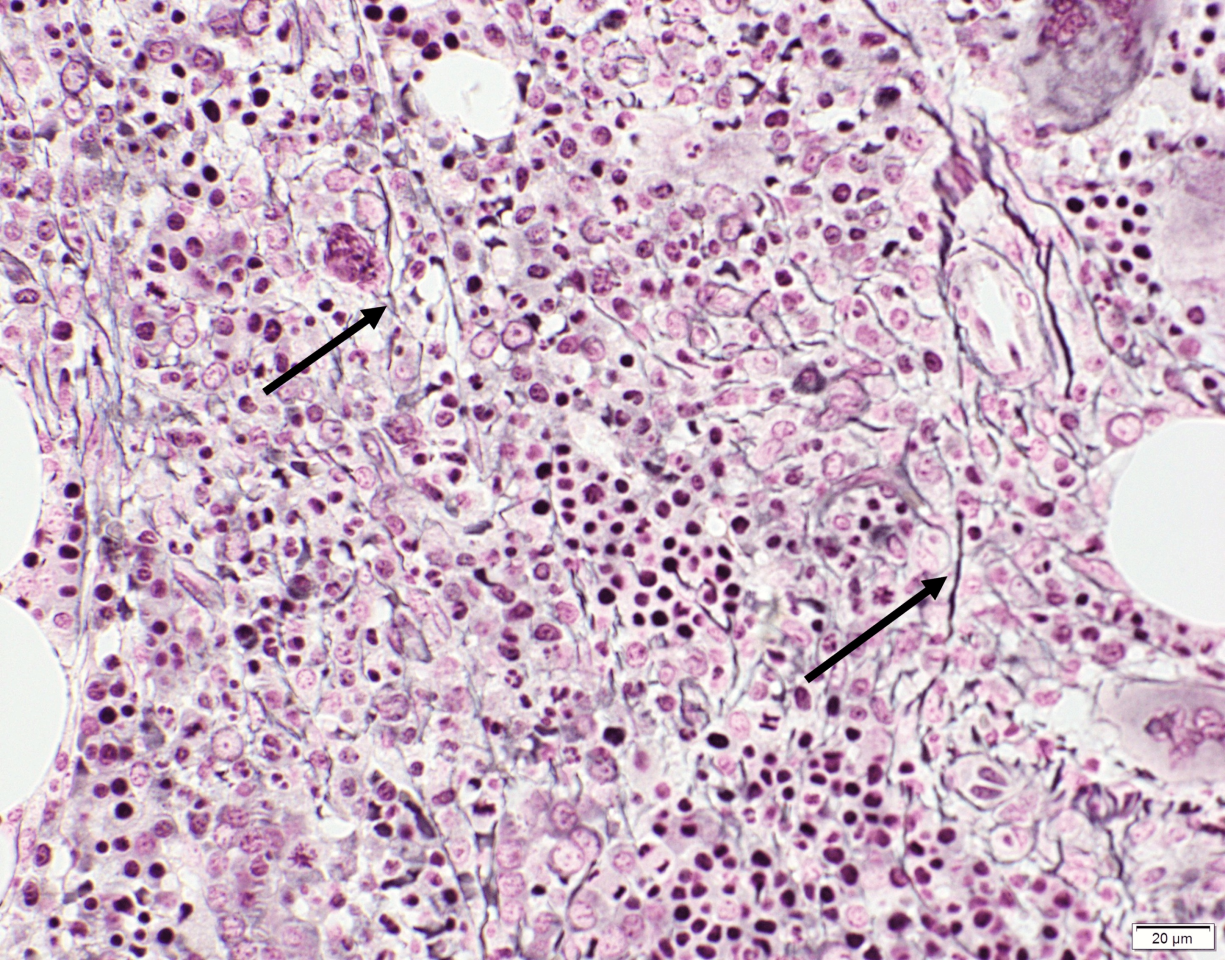
Mild reticulin fibrosis (MF-1) with a loose network of reticulin fibers shown by arrows. Reticulin staining, magnification ×400

During her admission, a bone marrow biopsy was performed to evaluate for myeloproliferative neoplasm given her history of positive CALR mutation. Histology (Figure 1) demonstrated hypercellular marrow. The myeloid to erythroid (M: E) ratio was estimated to be normal at 2:1. While an elevated M: E ratio is typical for pre-PMF, it was not elevated in this case. This was possibly due to reactive erythroid hyperplasia in response to the patient's known autoimmune hemolytic anemia. Reticulin stain (Figure 2) revealed mild reticulin fibrosis (MF grade 1). Given these findings, she was diagnosed with pre-PMF. Based upon her genetically inspired prognostic scoring system (GIPSS) score of 0, the patient was considered to be low-risk and placed on surveillance with routine lab work.

One year after hospitalization, the patient remains well. She has not required any further prednisone, IVIG, or rituximab. Her hemoglobin continues to remain within normal limits.

## Discussion

PMF is a rare chronic myeloproliferative disorder with an estimated prevalence of 4-6 per 100,000 adults between 2008 and 2010 in the United States [2]. This disease is thought to be due to abnormal proliferation of atypical megakaryocytes, which release pro-fibrotic and pro-inflammatory factors. As a result, fibroblasts produce excessive amounts of collagen which leads to bone marrow fibrosis [3]. Frequently, the only presenting findings are thrombocytosis and/or leukocytosis detected on routine lab work. Leukoerythroblastosis may also be seen, especially as the disease progresses. The diagnosis of myelofibrosis requires histologic evaluation of the bone marrow, which is characterized by granulocytic proliferation and abnormal megakaryocytic proliferation with progressive marrow fibrosis. There are two subtypes of myelofibrosis. Patients without any history of preceding myeloproliferative neoplasms are considered to have PMF, whereas myelofibrosis that evolves from pre-existing essential thrombocythemia or polycythemia vera is considered secondary myelofibrosis [4].

Approximately 90% of patients with PMF have a JAK2, CALR, or MPL mutation [1]. These genetic mutations are also linked with other myeloproliferative neoplasms such as essential thrombocythemia and polycythemia vera [5]. Risk stratification is performed using the GIPSS score, which determines risk using the presence or absence of certain mutations. In particular, an absence of CALR mutation (HT1) increases the risk score. In low-risk diseases, observation or symptom-directed therapy is recommended, whereas in high-risk diseases more aggressive treatments such as bone marrow transplant are necessary [6]. Clinically, patients with a CALR mutation tend to have a higher platelet count, lower leukocyte count, higher hemoglobin, and improved overall survival compared to those with a JAK2 mutation [7]. In addition, studies have shown that in patients with essential thrombocythemia and CALR mutation, there is no benefit from using low-dose aspirin, which is in contrast to patients with a JAK2 mutation. In these patients with CALR mutation, aspirin increases the risk of bleeding without decreasing the risk of thrombosis [8].

In 2016, The World Health Organization defined diagnostic criteria for early PMF, also known as pre-fibrotic PMF (pre-PMF). The diagnosis is made via bone marrow biopsy, with fibrosis grades of 0 or 1 classified as pre-PMF, while grades 2-4 are labeled as overt PMF [4]. It is estimated that there is a 36.9% risk of pre-PMF progressing to overt PMF over the course of 10 years [9]. Studies have shown that pre-PMF typically has a better prognosis compared to overt PMF, but worse than essential thrombocythemia [10].

The patient that we presented in this paper had an atypical initial presentation of PMF with wAIHA. Patients with wAIHA have immune system dysregulation, causing the production of autoantibodies (typically IgG) that bind to antigens on red blood cells at 37°C. This leads to accelerated destruction of these red blood cells. A DAT is typically positive in these cases. Approximately 50-60% of cases are associated with an underlying condition, with the remainder being idiopathic. These underlying conditions include infections, lymphoproliferative disorders (particularly chronic lymphocytic leukemia and multiple myeloma), autoimmune diseases, and immunodeficiencies [11]. wAIHA is typically treated with first-line steroids and IVIG, with rituximab commonly used as second-line treatment if the disease is refractory to steroids [12].

For our patient, we think that the etiology of her wAIHA is likely due to immune dysregulation secondary to PMF. Prior research has shown that cytokine dysregulation and autoantibody formation are commonly seen in PMF. Notably, a case series of 100 patients found positive mitogen-stimulated DAT (MS-DAT) despite negative standard DAT in 45% of patients with PMF. These patients likely had low-grade subclinical production of these autoantibodies, given similar hemoglobin/hemolysis labs when compared to patients with negative MS-DAT [7]. However, the presence of these autoantibodies likely predisposes patients to develop overt wAIHA. Cases of wAIHA have been seen with positive MS-DAT but negative DAT [13]. For this patient, we suspect that she initially had pre-existing autoantibodies with low-grade wAIHA, given her mild anemia and otherwise negative anemia workup at the time of initial presentation. As she was subsequently lost to follow-up for four years, it is unknown what caused the patient to progress to severe wAIHA requiring hospitalization. Further research is needed to investigate the mechanism for this formation of wAIHA antibodies as well as why the patient transformed into severe wAIHA.

## Conclusions

Underlying myelofibrosis should be considered as a rare etiology for a patient who presents with warm autoimmune hemolytic anemia and other hematologic abnormalities. This is likely related to cytokine dysregulation and autoantibody formation in PMF. Further research is needed to investigate the relationship between wAIHA and PMF, as well as the mechanism by which subclinical autoantibody formation transforms into overt wAIHA.
